# CCL20 chemokine and other proinflammatory markers after Ad26.COV2.S vaccination

**DOI:** 10.11613/BM.2024.030706

**Published:** 2024-10-15

**Authors:** Iva Ivanko, Milena Hanžek, Ivana Ćelap, Sandra Margetić, Domagoj Marijančević, Josipa Josipović, Petar Gaćina

**Affiliations:** 1 Department of Haematology, Sestre Milosrdnice University Hospital Center, Zagreb, Croatia; 2 Department of Clinical Chemistry, Sestre Milosrdnice University Hospital Center, Zagreb, Croatia; 3 Faculty of Pharmacy and Biochemistry, University of Zagreb, Zagreb, Croatia; 4 School of Medicine, Catholic University of Croatia, Zagreb, Croatia; 5 Department of Nephrology, Sestre Milosrdnice University Hospital Center, Zagreb, Croatia; 6 School of Dental Medicine, University of Zagreb, Zagreb, Croatia

**Keywords:** chemokine, vaccine, immunity

## Abstract

**Introduction:**

In highly stressed circumstances, such as COVID-19 pandemic, biomarkers of the vaccine-induced immunity could be especially convenient. The main aim of our study was to determine C-C motif ligand 20 (CCL20) concentration after Ad26.COV2.S vaccination in regard to more common proinflammatory molecules and its correlation with anti-SARS-CoV-2 antibody concentration. Secondly, we investigated inflammatory and immunologic profile differences between patients with and without arterial hypertension.

**Materials and methods:**

The study included 84 subjects vaccinated with Ad26.COV2.S vaccine. Concentration of CCL20, interleukin (IL) 6, C-reactive protein (CRP) was investigated before, 7 and 14 days after vaccination and concentration of anti-SARS-CoV-2 IgG antibody 7 and 14 days after vaccination. All the markers were measured by well-established laboratory methods.

**Results:**

There were no statistically significant changes of CCL20 and IL-6 concentration after the vaccination. The obtained results have shown statistically significant differences for CRP (P = 0.006) concentrations between 3 time points and SARS-CoV-2 IgG antibody (P < 0.001) concentrations between 2 time points. CCL20 did not correlate with IL-6, CRP or anti-SARS-CoV-2 IgG antibody concentration. Statistically significant difference for CRP (P = 0.025) concentration between 3 time points was observed in the subgroup of subjects with arterial hypertension.

**Conclusions:**

Although our results did not show changes in CCL20 concentration after the vaccination, possibly due to the study timeframe, further investigations on chemokine profile post SARS-CoV-2 immunization could facilitate the recognition of specific patterns of response (supra- or sub-optimal) to the vaccine.

## Graphical abstract

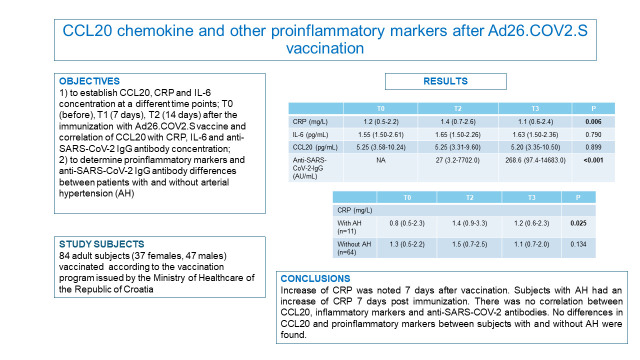

## Highlights

• Increase of C-reactive protein (CRP) was noted 7 days after vaccination

• Increase of CRP was noted 7 days after vaccination in subjects with arterial hypertension

• No correlation between C-C motif ligand 20 (CCL20), inflammatory markers and anti-SARS-COV-2 antibodies

• No differences in CCL20 and proinflammatory markers between subjects with and without arterial hypertension

## Introduction

Chemotactic cytokines (chemokines) are signaling molecules whose primary function is the recruitment of leukocytes and other inflammatory cells from the intravascular space, across the epithelium and endothelium to the specific sites within tissues during various inflammatory conditions (*1*). Both cytokines and chemokines are important drivers of the innate immunity and supporters of the adaptive immunity development.

Chemokine serum elevation has been documented and described after various vaccination (yellow fever, HIV, *etc.*) (*2*, *3*). An increase in chemokine/cytokine profile was also observed after immunization with severe acute respiratory syndrome coronavirus 2 (SARS-CoV-2) vaccines (*4*-*6*). This implies that such molecules could be potentially used as a biomarker of the vaccine-induced immunity and facilitate the recognition of individuals with supra- or sub-optimal responses, which could be especially convenient during pandemic.

C-C motif ligand 20 (CCL20), a chemokine expressed by activated macrophages, is responsible for the recruitment of the proinflammatory helper and regulatory T-cells and is a common chemotactic molecule for both coronavirus disease 2019 (COVID-19) and multisystem inflammatory syndrome in children (MIS-C) (*7*). CCL20 belongs to the subgroup of chemokines who are expressed constitutively but can be further induced in response to various cytokines and persist several days or longer (*8*). Some therapeutical approaches, such as interferon therapy in chronic hepatitis C can cause elevation of the CCL20, lasting several weeks, which is why CCL20 was used as a prognostic marker during interferon therapy (*9*).

Together with other proinflammatory biomarkers such as interleukin (IL-6) and C-reactive protein (CRP) it could potentially elucidate the level of immune response to the SARS-CoV-2 vaccine. To the best of our knowledge, no prior study has investigated CCL20 and its possible relationship with antibody concentration *post* Ad26.COV2.S immunization. We hypothesized that Ad26.COV2.S vaccine will induce CCL20 together with CRP and IL-6 expression as a part of an immunologic reaction to the vaccination which will correlate with an anti-SARS-CoV-2 antibody concentration. On the grounds of prior studies of the blood pressure changes after SARS-CoV-2 immunization, we were also interested in determining possible differences in inflammatory and immunologic response amongst subjects with and without arterial hypertension (AH) (*10*). Based on that idea, we conducted a prospective observational trial with the following aims: 1) to establish CCL20, CRP and IL-6 concentration at different time points after the immunization with Ad26.COV2.S (Janssen, Beerse, Belgium) vaccine and correlation of CCL20 with CRP, IL-6 and anti-SARS-CoV-2 IgG antibody concentration; 2) to determine proinflammatory markers and anti-SARS-CoV-2 IgG antibody differences between patients with and without AH.

## Materials and methods

### Subjects

This prospective observational study included 84 adult subjects who were vaccinated with recombinant vector Ad26.COV2.S vaccine from April to August 2021 at the University Hospital Centre Sestre milosrdnice and Medical Centre Centar, Zagreb, Croatia, according to the vaccination program issued by the Ministry of Health of the Republic of Croatia. Study subjects were volunteers chosen at the vaccination site by the head investigator. Of the initially 150 subjects, 84 of them were included according to the main criteria of the study.

The main exclusion criteria were active malignant disease, recent major surgery (one month before vaccination), thromboembolic incident 3 months prior to the vaccination, dialysis, peripheral artery disease, diabetes requiring insulin therapy, active immunologic disorder, pregnancy, puerperium, oral contraceptive therapy, anticoagulant therapy, use of nonsteroid antirheumatic therapy, immune thrombocytopenia and hemophilia. Subjects with acute infection of any kind were excluded by the vaccination criteria. The study participants were also a part of a larger study which investigated hemostasis and proinflammatory markers *post* SARS-CoV-2 vaccination, hence the vast exclusion criteria (*11*).

Subjects with a confirmed prior diagnosis of COVID-19 disease were not excluded from the study since the elapsed time between SARS-CoV-2 infection and vaccination was at least 6 months (in alignment with the official recommendations of the Croatian Institute of Public Health).

Data on body mass index (BMI), presence of AH and other comorbidities were collected upon study entry.

The study included measurement of CCL20, IL-6 and CRP concentration at 3 different time points for each participant: before vaccination (within 24 hours), 7 and 14 days after immunization. The anti-SARS-CoV-2 IgG concentration was measured 7 and 14 days after immunization in order to correlate inflammatory and immunologic response to the vaccination. The study participants were obligated to report symptoms of a possible acute infection of any kind (fever, malaise, rash, *etc.*) after vaccination (at each sequential venipuncture) as this would interfere with the study results and lead to the trial exclusion. Additionally, the subjects were followed up to 6 months *post* vaccination for the possible occurrence of blood pressure changes.

The study was performed according to the guiding principles of the Declaration of Helsinki and was approved by the University Hospital Centre and Medical Centre’s Ethic committees (EP-251-29-11-21-01-10; EP-251-510-03-20-21-09). Informed consent was obtained from all participants included in the study.

### Methods

All laboratory tests were performed in the Department of Clinical Chemistry at the University Hospital Centre Sestre milosrdnice. Blood was collected in Vaccuette test tubes (Greiner Bio-One, Kremmsmünster, Austria) without anticoagulant and centrifuged for 10 minutes on 1800xg within 3 hours after venipuncture. Determination of CRP was done immediately while the samples of serum for IL-6, CCL20 and quantitative SARS-CoV-2 IgG concentration were stored at - 80°C until they were analyzed.

Measurement of CRP concentration was performed using original manufacturer’s reagents on Architect c8000 clinical chemistry analyzer (Abbott Diagnostics, Abbott Park, USA). Measurement of IL-6 with a non-competitive (sandwich) electrochemiluminescent immunoassay (ECLIA) was performed using original manufacturer’s reagents on Roche Cobas analyzer e801 (Roche Diagnostics GmbH, Mannheim, Germany). Concentration measurement of the quantitative anti-SARS-CoV-2 IgG antibody against the spike receptor-binding domain (RBD) of SARS-CoV-2 with chemiluminescent microparticle immunoassay (CMIA) was performed using original manufacturer’s reagents on Alinity i system (Abbott Laboratories, Abbot Park, USA). Concentration of CCL20 was measured using Human MIP-3 alpha/CCL20 PicoKine ELISA Kit (MyBioSource Cat# MBS175983). This enzyme-linked immunosorbent assay (ELISA) kit is a solid-phase immunoassay that captures human CCL20 on a pre-coated strip plate with a monoclonal antibody. The detection antibody is a polyclonal biotinylated antibody which after reaction with avidin-biotin-peroxidase complex and color developing reagent produces a blue color product that changes into yellow after adding an acidic stop solution. The absorbance of the yellow product at 450 nm is linearly proportional to human CCL20 in the sample. Measuring range of the test is 7.8-500 pg/mL. According to the manufacturer, both the intra- and inter-assay precision is 6.9% at a concentration of 78 pg/mL and 73 pg/mL respectively.

### Statistical analysis

The distribution of the data was tested by Kolmogorov-Smirnov test. Since the normality of the data distribution failed, the results are presented as median and interquartile range (IQR) and non-parametric statistics is used for statistical calculations were appropriate. Age is presented as median and range (minimum-maximum). Categorical variables are presented as counts and proportions. To test the differences between results obtained at different time points (3 points), we used Friedman test. Differences between results obtained for SARS-CoV-2 antibodies concentrations 7 and 14 days after vaccination was tested with Wilcoxon paired test. Differences in results between two independent groups (subjects with and without AH) were tested with Mann-Whitney test. Correlation of the measured parameters results was tested using non-parametric Spearman rank correlation test. The value of P *<* 0.05 was considered statistically significant. All statistical tests were done using MedCalc Statistical Software version 20.010 (MedCalc Software Ltd., Ostend, Belgium; https://www.medcalc.org; 2021).

## Results

Demographic characteristics of the studied patients are presented in Table 1. Median age was 44 years and the study included 37 (0.44) females out of 84 subjects. Only 11 (0.13) subjects suffered from AH, while 11 (0.13) subjects had other chronic illness (diabetes type 2 (N = 3), epilepsy (N = 1), psoriasis (N = 1), asthma (N = 2), hyperthyroidism (N = 2), hyperlipidemia (N = 2)).

**Table 1:** Demographic characteristics of the studied subjects

	**N = 84**
Age, years	44 (19-66)
Sex, female (N, proportion)	37 (0.44)
Sex, male (N, proportion)	47 (0.56)
BMI (kg/m^2^)	25.3 (22.6-28.7)
Arterial hypertension (N, proportion)	11 (0.13)
Chronic illness (N, proportion)	11 (0.13)
Age is presented as median (range). BMI - body mass index.	

The obtained results have shown statistically significant differences for CRP (P = 0.006) concentration between three time points. *Post hoc* analysis pointed that the CRP concentration is statistically higher 7 days after vaccination *vs* before and 14 days after vaccination. Also, the results showed statistically significant differences for anti-SARS-CoV-2 IgG antibody (P < 0.001) concentration between two time points. There were no statistically significant differences between different time points after vaccination for the concentrations of IL-6 and CCL20 (Table 2). No correlation between CCL20, inflammatory markers and anti-SARS-COV-2-IgG antibody was established (Table 3). There were no differences between studied proinflammatory markers and SARS-CoV-2 antibodies in the subjects with and without AH. Subjects with AH showed statistically significant differences for CRP (P = 0.025) concentration between three time points (*post hoc* analysis resulted in statistically lower CRP concentrations before vaccination *vs* 7 and 14 days after vaccination). Also, SARS-CoV-2 IgG antibody concentration in subjects with and without AH was statistically higher 14 days after vaccination *vs* 7 days after vaccination (P = 0.003; P < 0.001) (Table 4).

**Table 2:** Proinflammatory markers and anti-SARS-CoV-2 IgG antibody differences between different time points after vaccination in the studied subjects

**Parameter**	**Median (IQR)**	**P**
CRP (mg/L)		
T0	1.2 (0.5-2.2)	0.006
T1	1.4 (0.7-2.6)	
T2	1.1 (0.6-2.4)	
IL-6 (pg/mL)		
T0	1.55 (1.50-2.61)	0.790
T1	1.65 (1.50-2.26)	
T2	1.63 (1.50-2.36)	
CCL20 (pg/mL)		
T0	5.25 (3.58-10.24)	0.899
T1	5.25 (3.31-9.60)	
T2	5.20 (3.35-10.50)	
Anti-SARS-CoV-2 IgG (AU/mL)		
T0	NA	< 0.001
T1	27.3 (3.2-7702.0)	
T2	268.6 (97.4-14683.0)	
CRP, IL-6 and CCL2 differences were tested using the Friedman test. Differences in anti-SARS-CoV-2 IgG were tested using the Wilcoxon test. *Post-hoc* analysis for CRP: the CRP concentration is statistically higher 7 days *post* vaccination *vs* before vaccination and 14 days *post* vaccination (P < 0.05). P < 0.05 was considered statistically significant. CCL20 - chemokine (C-C motif) ligand 20. CRP - C- reactive protein. IL6 - interleukin 6. T0 - before vaccination. T1 - 7 days after vaccination. T2 - 14 days after vaccination. NA - not applicable.		

**Table 3:** Correlation of CCL20 and proinflammatory markers and anti-SARS-CoV-2 IgG antibodies measured at different time points

**Marker/time point**	**CCL20** **T0**	**CCL20** **T1**	**CCL20** **T2**
CRP T0	0.013 P=0.908	/	/
CRP T1	/	0.045 P=0.692	/
CRP T2	/	/	0.095 P=0.390
IL-6 T0	0.450 P=0.722	/	/
IL-6 T1	/	0.161 P=0.153	/
IL-6 T2	/	/	0.111 P=0.318
anti-SARS-CoV-2 IgG T1	/	- 0.066 P=0.562	/
anti-SARS-CoV-2 IgG T2	/	/	- 0.084 P=0.450
Spearman test was used to test the correlation. P < 0.05 was considered statistically significant. CCL20 - chemokine (C-C motif) ligand 20. CRP – C - reactive protein. IL-6 - interleukin 6. T0 - before vaccination. T1 - 7 days after vaccination. T2 - 14 days after vaccination.			

**Table 4:** Proinflammatory markers and anti-SARS-CoV-2 IgG antibody differences between patients with and without AH

**Marker/time point**	**With AH (N = 11)**	**Without AH (N = 64)**	**P**
**CRP (mg/L)**			
T0	0.8 (0.5-2.3)	1.3 (0.5-2.2)	0.735
T1	1.4 (0.9-3.3)	1.5 (0.7-2.5)	0.828
T2	1.2 (0.6-2.3)	1.1 (0.7-2.0)	0.839
P	0.025	0.134	
**IL-6 (pg/mL)**			
T0	1.50 (1.50-2.59)	1.55 (1.50-2.61)	0.891
T1	1.65 (1.50- 2.33)	1.66 (1.50-2.26)	0.795
T2	1.78 (1.50-2.42)	1.55 (1.50-2.31)	0.829
P	0.506	0.663	
**CCL20 (pg/mL)**			
T0	6.10 (2.50-10.64)	5.46 (3.60 - 11.73)	0.729
T1	5.63 (4.11-10.96)	5.36 (3.26 - 9.66)	1.000
T2	5.29 (4.11-11.18)	4.92 (3.35-11.07)	0.770
P	0.905	0.919	
**anti SARS-CoV-2 antibodies (AU/mL)**			
T1	3729.3 (2.3-10071.1)	23.9 (2.9-5994.5)	0.928
T2	6066.1 (74.3-14683.2)	232.7 (77.7-7931.4)	0.716
P	0.003	< 0.001	
CRP, IL-6 and CCL2 time point differences were tested using the Friedman test. Time point differences in anti-SARS-CoV-2 IgG were tested using the Wilcoxon test. Differences between subjects with and without AH were tested using the Mann-Whitney test. *Post-hoc* analysis for CRP: the CRP concentration was statistically lower before vaccination *vs* 7 and 14 days *post* vaccination (P < 0.05). P < 0.05 was considered statistically significant. CCL20 - chemokine (C-C motif) ligand 20. AH – arterial hypertension. CRP – C - reactive protein. IL-6 - interleukin 6. T0 - before vaccination. T1 - 7 days after vaccination. T2 - 14 days after vaccination.			

## Discussion

The CRP concentration showed statistically significant changes *post* vaccination with the highest concentration 7 days after immunization. The TREASURE study by Brambilla *et al.*, which enrolled a significant number of subjects (N = 368), found an increase in the CRP concentration 8 ± 2 days *post* immunization regardless of the vaccine type (BNT162b2, mRNA-1273, ChAdOx-1, Ad26.COV2.S) (*12*). This is consistent with a well-known postvaccination reaction, stated also by the vaccine manufacturer (*13*).

Clinical safety investigation of Ad26.COV2.S vaccine observed a more prominent local and systemic postvaccination inflammatory reaction in younger participants (18-55 years) (*14*). Median age of our study subjects was 44 years, majority of which had a mild reaction to the vaccination which can explain statistically significant change in the CRP concentration.

Our results show no changes in CCL20 concentration in subjects after Ad26.COV2.S vaccination. Unfortunately, there are no previously described similar studies on CCL20 after SARS-CoV-2 vaccination. However, a rise of similar chemokine profile has been documented after various SARS-CoV-2 vaccines. Bergamaschi *et al.* compared cytokine and chemokine responses after vaccination with BNT162b2 mRNA (Pfizer/BioNtech) vaccine (*4*). Their results showed an increase of C-X-C motif chemokine 10 (CXCL10) within 48 h after vaccination, while increased concentration of C-C motif ligands 4 (CCL4) persisted 8 days *post* immunization. The chemokine increase correlated with Spike antibody concentration, supporting their role as markers of humoral immunity development.

Lee *et al.* found a CXCL10 increase within a week of ChAdOx1 vaccination (*6*). Kumar *et al.* demonstrated an increase of various chemokines *post* BBV152 immunization (*5*). While some chemokines, such as CXCL10 were decreased by the end of the first week *post* immunization, concentration of others, such as CCL4, stayed elevated for months.

Based on the previous knowledge on CCL20, we hypothesized an elevation of CCL20 concentration after Ad26.COV2.S vaccination as a part of immunologic reaction to the SARS-CoV-2 vaccine. One plausible explanation for the lack of CCL20 increase in our study is a missed window of increase of this particular chemokine, closer to the vaccination.

IL-6 concentration after SARS-CoV-2 vaccination has also been studied during pandemic with the aim of establishing an immunological response to the vaccine stimuli. The aforementioned TREASURE study confirmed an increase in IL-6 concentration 8 ± 2 days after both mRNA and vector-based SARS-CoV-2 vaccine exposure (*12*). Interestingly, our study did not show changes in the IL-6 concentration which would be expected considering a similar study timeframe and even larger subject pool (in the TREASURE study the number of subjects vaccinated with Ad26.COV2.S was relatively small, N = 19).

To the best of our knowledge, there are no published studies on IL-6 changes *post* Ad26.COV2.S vaccination to compare with our results. However, we also considered trials with other adenoviral vaccines. Ostrowski *et al.* found a more prominent increase of IL-6 together with several inflammatory markers 8-16 days (median 11 days) *post* ChAdOx1-S vaccination *vs* BNT162b2 /mRNA-1273, which led to the conclusion of a stronger inflammatory response after adenoviral vaccine (*15*). The downside of the study is the lack of clearly stated exclusion criteria (if there were any). Obviously, there are various physiologic or pathologic processes which could influence the results and cause IL-6 to increase.

The lack of change in IL-6 in our study may be due to the missed early increase (1-2 days *post* vaccination) which was presented in the study of Bergamaschi *et al* (*4*). Timeframe of our study was formed on the basis of the scientific evidence on vaccine induced thrombotic thrombocytopenia (VITT) presentation, usually 5-30 days *post* SARS-COV-2 immunization (*16*). However, it should be noted that the studied vaccine by Bergamaschi *et al.* was of the mRNA design (BNT162b2).

Our study had multiple exclusion criteria, as previously stated, in order to minimize biases which could potentially undermine the results. We did not exclude arterial hypertension due to the lack of a direct impact on the inflammatory markers. The participants with AH were in a minority (N = 11). However, due to the emerging scientific evidence of the impact of SARS-CoV-2 vaccination on the arterial pressure, we looked for the potential differences in the inflammatory and immunologic response after SARS-CoV-2 immunization based on the history of AH (*10*). The subjects with AH experienced a rise in the CRP concentration after vaccination. There were no changes in the concentration of IL-6 and CCL20 between patients with/without AH. Subjects with and without AH had higher IgG antibody concentration after vaccination, which was expected (P = 0.003; P < 0.001) (Table 4). None of the subjects, with or without AH, reported a blood pressure disruption in the 6 months period *post* immunization.

Due to the small number of subjects with AH in our study, it would be bold to presume that the participants with AH are more prone to the proinflammatory events after SARS-CoV-2 vaccination on the basis of preexisting endothelial damage. Future well conducted investigations with a larger number of study participants could potentially elucidate the effect of SARS-CoV-2 vaccine on the blood pressure.

In our study, clearly, there is a rise in the concentration of anti-SARS-CoV-2 IgG antibodies *post* immunization (statistically higher concentration was noted 14 days *vs* 7 days after vaccination (P < 0.001)). This was expected and in line with the results of clinical study on safety and efficacy of single-dose Ad26.COV2.S vaccine which demonstrated a high protection against moderate to severe COVID-19 disease 14 days after the immunization (*17*).

Lack of correlation between CCL20 and IL-6, CRP and anti-SARS-CoV-2 IgG antibody can also be explained by the missed early rise of CCL20 chemokine. Presumably, an additional time point, closer to the vaccination (24-72 hours after immunization) would potentially demonstrate a rise in all three proinflammatory markers.

Our study has several limitations. First of all, number of study participants is rather small, which could have been an issue when dividing subjects into subgroups based on their characteristics (AH). Second, due to the timeframe of the study, a potential rise in parameters could have been missed. The study would have benefited with an additional venipuncture closer to the immunization. Finally, statistically significant changes in CRP concentration after vaccination are clinically irrelevant.

In conclusion, the results of our study show an increase in the CRP concentration 7 days after Ad26.COV2.S vaccination, which is in alignment with previously known scientific evidence. We did not observe an increase of IL-6 or CCL20 concentration after vaccination. There was no correlation between CCL20 concentration and other proinflammatory markers and anti-SARS-CoV-2 antibodies. This could potentially be the result of the timeframe in which the laboratory measurements were conducted. Further investigations with a several time points closer to the vaccination are needed to shed more light on the significance of CCL20 after SARS-CoV-2 immunization.

## Data Availability

All data generated and analyzed in the presented study are included in this published article
